# Bioinspired Young's Modulus‐Hierarchical E‐Skin with Decoupling Multimodality and Neuromorphic Encoding Outputs to Biosystems

**DOI:** 10.1002/advs.202304121

**Published:** 2023-09-07

**Authors:** Shengshun Duan, Xiao Wei, Fangzhi Zhao, Huiying Yang, Ye Wang, Pinzhen Chen, Jianlong Hong, Shengxin Xiang, Minzhou Luo, Qiongfeng Shi, Guozhen Shen, Jun Wu

**Affiliations:** ^1^ Joint International Research Laboratory of Information Display and Visualization School of Electronic Science and Engineering Southeast University Nanjing 210096 China; ^2^ Jiangsu Jitri Intelligent Manufacturing Technology Institute Co., Ltd. Photoelectric technology park of Jiangbei New District Nanjing 211500 China; ^3^ School of Integrated Circuits and Electronics Beijing Institute of Technology Beijing 100081 China

**Keywords:** bioinspired, electronic skin, human‐prosthetics interfaces, machine learning, multimodal sensors, neural models

## Abstract

As key interfaces for the disabled, optimal prosthetics should elicit natural sensations of skin touch or proprioception, by unambiguously delivering the multimodal signals acquired by the prosthetics to the nervous system, which still remains challenging. Here, a bioinspired temperature‐pressure electronic skin with decoupling capability (TPD e‐skin), inspired by the high‐low modulus hierarchical structure of human skin, is developed to restore such functionality. Due to the bionic dual‐state amplifying microstructure and contact resistance modulation, the MXene TPD e‐skin exhibits high sensitivity over a wide pressure range and excellent temperature insensitivity (91.2% reduction). Additionally, the high‐low modulus structural configuration enables the pressure insensitivity of the thermistor. Furthermore, a neural model is proposed to neutrally code the temperature‐pressure signals into three types of nerve‐acceptable frequency signals, corresponding to thermoreceptors, slow‐adapting receptors, and fast‐adapting receptors. Four operational states in the time domain are also distinguished after the neural coding in the frequency domain. Besides, a brain‐like machine learning‐based fusion process for frequency signals is also constructed to analyze the frequency pattern and achieve object recognition with a high accuracy of 98.7%. The TPD neural system offers promising potential to enable advanced prosthetic devices with the capability of multimodality‐decoupling sensing and deep neural integration.

## Graphical abstract

To promote the development of constructing a direct neural link between prosthetics and the disabled, a bioinspired neuromorphic e‐skin is proposed to produce nerve‐acceptable frequency spikes with biological plausibility for pressure and temperature stimuli without mutual interference. Besides, the signal patterns in the frequency domain after neural coding are proven to be effective for manipulation states and contacted object cognition.

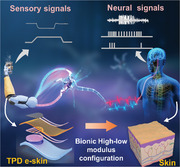

## 1 Introduction

Prosthetics that can improve the quality of life of amputees have been the hot spot in the past decades. Nowadays, commercial prosthetics are getting ever more sophisticated. However, they do not provide natural sensory information on the interaction with objects or movements. The inherent disadvantages include unphysiological manipulation of prosthetics and difficulty in controlling the force exerted with prosthetics, thus leading to the absence of ownership and possibly creating health issues.^[^ ^1^ ^‐^ ^3^ ^]^

Therefore, restoring natural sensory feedback from the prosthetics to amputees is an unmet clinical need. Electronic skin (e‐skin) inspired by human skin is stretchy and able to respond to touch, offering options for sensory prosthetics.^[^ ^4^, ^5^, ^6^, ^7^, ^8^ ^]^ Traditional e‐skin often provides haptic feedback through skin‐integrated actuators or electro‐tactile techniques.^[^ ^9^, ^10^, ^11^ ^]^ Yet, it is not intuitive, not nerve‐acceptable, and requires the brain to perform secondary signal decoding. An optimal prosthetics should be able to elicit natural sensations of touch or proprioception, by delivering the complex signals acquired by the prosthetics to the nervous system. Frequency encoding of touch stimuli through hardware and computational approaches has been proven to be nerve‐acceptable.^[^ ^12^, ^13^, ^14^ ^]^ For example, Bao et al. proposed an organic transistor circuit that transduces pressure into digital frequency signals directly, which is further used to stimulate optogenetically engineered mouse somatosensory neurons of mouse cortex in vitro, achieving stimulated pulses in accordance with pressure.^[^ ^12^ ^]^ Yet, the reported neuromorphic prosthetics mainly focused on sensing and neural coding of pressure. It remains a challenge to produce the frequency spikes with biological plausibility for multimodal mechanical/environmental stimuli (temperature, pressure, etc.) without mutual interference. Moreover, for complex tasks such as manipulation states and contacted object cognition of the prosthetics, whether its patterns in the frequency domain after neural coding are suitable for neural analysis or not has been not explored and verified.

Among these multimodal stimuli, the senses of heat, cold, and pressure, via thermoreceptors, slow‐adapting (SA) receptors, and fast‐adapting (FA) receptors, are the most essential and significant for survival, which underpins interactions with the world around humans.^[^ ^15^, ^16^, ^17^, ^18^ ^]^ Early research on multimodal temperature‐pressure e‐skin mainly focuses on achieving the response to pressure and temperature simultaneously based on the multi‐stimuli response ability of functional conductive nanomaterials (i.e., graphite, carbon nanotubes, carbon black, MXene).^[^ ^19^, ^20^, ^21^, ^22^, ^23^ ^]^ Yet, dual signals acquired from a simply integrated device are mixed and overlapped, making it difficult to decouple the temperature and pressure signals independently.

Nowadays, many studies have been devoted to decoupling temperature and pressure signals. These devices monitor temperature using intrinsic pressure‐insensitive thermoelectric and pyroelectric effects and detect pressure based on piezo‐resistive and ‐capacitive effects.^[^ ^24^, ^25^, ^26^, ^27^ ^]^ Yet, these devices have inherent disadvantages: 1) a relatively low‐temperature sensitivity due to the low Seebeck/pyroelectric coefficient of soft materials; 2) not capable to obtain the absolute temperature value; 3) complication of information acquisition and processing system due to different sensing mechanism and output signals; 4) complex preparation processes. These challenges hinder their adaptabilities in applications that require accurate temperature‐pressure acquisition in a compact system.

Piezoresistive sensors and thermistors are currently the most prospective technologies for pressure and temperature sensing, respectively. They both operate under the mechanism of resistance change, which could have the intrinsic advantage to overcome the above issues of multimodal sensors, due to their similar signal patterns and manufacturing process.^[^ ^28^ ^]^ Yet, how to rationally design the structure for multimodal information sensing and decoupling based on these two mechanisms remains a challenge. On one hand, similar to the hierarchical structure of the skin, the vertical hierarchical assembly of different sensing structures has been proven effective for constructing multimodal sensors.^[^ ^29^, ^30^, ^31^, ^32^ ^]^ In the hierarchical assembly, skin‐inspired microstructure engineering and vertically hierarchical strategy are employed to enhance pressure‐sensing sensitivity. Abrasive structures and leaf pattern structures are the most used microstructures to simulate the random Gaussian distribution (RGD) spinosum microstructures under the epidermis, which has been proven to improve pressure sensitivity.^[^ ^33^, ^34^, ^35^, ^36^ ^]^ However, these random microstructures with limited height difference lead to rapid pressure saturation, thereby limiting their application in broad pressure‐range scenarios.^[^ ^28^ ^]^ On the other hand, independent decoupling of multimodal stimuli for bionic hierarchical multimodal sensors through skin‐inspired structural optimization is rarely reported. The accuracy of thin‐film temperature sensors would be affected by pressure‐induced deformation. Alternatively, the active layer in the pressure sensors, which is made of conductive nanomaterials, also reacts to temperature changes, affecting the accuracy of the pressure measurement.^[^ ^37^, ^38^ ^]^ Therefore, developing resistive‐type dual‐parameter sensors with decoupled pressure‐temperature reading, superior sensitivity, wide range, and a simple preparation process requires further research efforts. In addition, the simultaneous detection, acquisition, transmission, and post‐processing of complex bimodal tactile information within a compact system require a new wireless sensing system design, not relying on commercial measuring instruments.

Herein, inspired by the hierarchical structure with high‐low modulus configuration of the human skin, we present a bimodal temperature‐pressure e‐skin with decoupling capability via a simple screen‐printing technique and layer‐by‐layer assembly. Cast from the bark of plane trees, the bionic two‐stage amplifier microstructures enable both high sensitivity and wide sensing range of the pressure sensor (795 kPa^−1^ at 30–50 kPa and 1319 kPa^−1^ at 100–300 kPa), inhibiting the rapid pressure saturation of traditional spinosum‐like RGD microstructures. Since the pressure response of the contact resistance sensor is mainly determined by the resistance of the contact interface formed by the conductive functional material and the electrodes, the contact resistance modulation of the pressure sensor is inherently not sensitive to temperature, reducing the temperature response by 91.2%. Thereby, the confounding temperature effect is minimized. In addition, the high‐low modulus structural configuration formed by configuring the soft Ecoflex layer under the pressure sensor enables the pressure insensitivity of the thermistor. Moreover, we design a customized signal processing and wireless transmission module, which liberates the TPD e‐skin from bulky measuring instruments and enables versatile robotic applications. To interface with the biological systems directly, a neural model is used to neurally code the bimodal temperature‐pressure signals into three‐type nerve‐acceptable frequency signals, corresponding to thermoreceptors, SA, and FA receptors. As a demonstration, after neural coding, four operational states of a robotic hand in the time domain are also distinguished in the frequency domain. Moreover, as a proof‐of‐concept, a brain‐like fusion process based on an artificial neural network for neurally coded frequency signals is developed, which could recognize 15 kinds of objects with an accuracy of 98.7%. This TPD e‐skin system could find more applications in advanced intelligent prosthetics, human‐robotic interfaces, human enhancement for people with disabilities, and embodied interaction in Metaverse applications.

## 2 Results and Discussion

**Figure 1:**
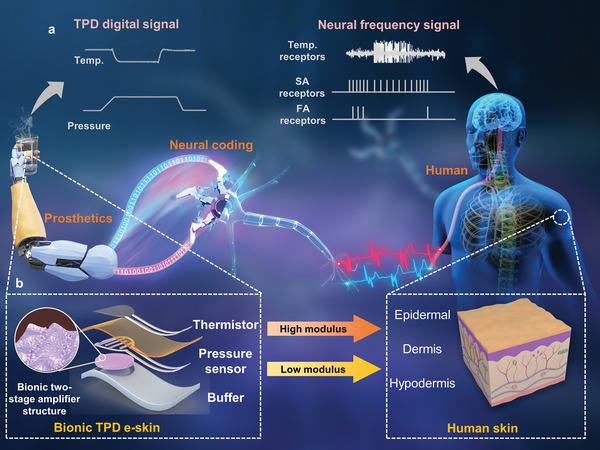
Schematic illustration of the proposed TPD e‐skin system. a) The TPD e‐skin system that can sense temperature‐pressure stimuli and code them into nerve‐acceptable frequency signals to interface with the biological systems directly. b) The TPD e‐skin exhibits a vertically hierarchical structure with a high‐low modulus configuration, like the human skin, of which the pressure sensors feature a bionic two‐stage amplifier structure.

**Figure** **1**a shows the schematic of human‐prosthetics interfaces. The TPD e‐skin systems attached to the prosthetics arm and fingers could sense the pressure and temperature stimuli independently. This bimodal information is then neurally coded into three types of nerve‐acceptable frequency signals, which could directly interface with the biological systems. Mimicking the vertically hierarchical structure of the human skin, the TPD e‐skin features a vertically hierarchical structure, high‐low modulus configuration, and a dual‐state amplifying microstructure, which enables the TPD e‐skin high‐pressure sensitivity over a wide range and decoupling ability of temperature and pressure (Figure 1b).

### 2.1 TPD E‐Skin with a Skin‐Like Bionic Microstructure

Nature often inspires engineering development, especially for artificial electronic devices with various bionic structures. For example, the skin is the most critical tactile‐sensing organ in the human body. The spinosum layer under the epidermis and sweat hair with various heights both enhance the pressure perception of the skin.^[^ ^28^ ^]^ In addition, the epidermis, dermis, and hypodermis in the human skin feature different Young's modulus, playing different roles in functions. The Young's modulus of the epidermis is ≈4 MPa at 4–10 kHz, which could protect humans from the outside world. Young's moduli of the dermis and hypodermis are ≈40 and 15 kPa at 0.2–1 kHz, respectively, which contain stimuli‐sensing receptors and nerve endings.^[^ ^39^, ^40^ ^]^

**Figure 2:**
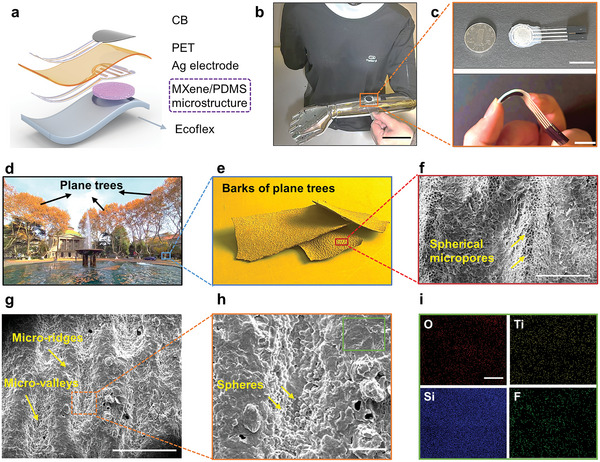
Structure and characterizations of the TPD e‐skin. a) Schematic diagram of the TPD e‐skin comprised of a CB thermistor, a pressure sensor, and a low‐modulus Ecoflex layer. b) Photograph of the TPD e‐skin at the robotic arm. Scale bar: 10 cm. c) Photograph of the TPD e‐skin (top), which is bendable (bottom). Scale bar: 3 cm. d) Photograph of the plane trees in Southeast University. e) The bark of plane trees. f) SEM image showing surface structures of the bark of plane trees, with spherical micropores in micro‐ridges. Scale bar: 500 µm. g) SEM image showing surface structures of PDMS that were cast from the bark of plane trees, with continuous micro ridge‐valley structures. Scale bar: 1 mm. h) SEM image of the microspheres in the micro‐valleys. Scale bar: 80 µm. i) Elemental maps of O, Ti, Si, and F elements of the MXene‐coated PDMS. Scale bar: 20 µm.

Therefore, a skin‐like hierarchical structure featuring a high‐low modulus configuration and a dual‐state amplifying microstructure is proposed to develop a multimodal robust integrated sensing e‐skin with reduced signal crosstalk between different sensors. **Figure** **2**a shows the structure of the TPD e‐skin, which integrates a CB thermistor, a high‐performance pressure sensor with the bionic dual‐state amplifying microstructure, and a low‐modulus Ecoflex buffer into a single stack. Specifically, the upper polyethylene terephthalate (PET) layer hosting the thermistor and pressure sensor electrode presents a high Young's modulus of ≈5 GPa while the lower pressure‐sensitive micro‐structured polydimethylsiloxane (PDMS) layer and the Ecoflex buffer layer have low Young's moduli of ≈3 MPa and ≈60 kPa, respectively. More details of the fabrication process of the TPD e‐skin are provided in the Experimental Section. The prepared TPD e‐skin exhibits good mechanical performance, which can be integrated at the robotic arm and bent easily (Figure 2b,c).

The CB ink with good thermal response was screen‐printed on the PET film to form the CB thermistor. Then, Ag ink was screen‐printed on the PET film as the conductive interface between the CB thermistor and soft Dupont lines, and also the backside of the PET film as the electrode of the pressure sensor, with the optical images depicted in Figure S1 (Supporting Information). The pressure sensor is composed of a PET film, an Ag interdigitated electrode layer, an adhesive layer, and an MXene‐coated micro‐structured PDMS layer from top to bottom. Among them, the microstructures were cast from the bark of plane trees (Latin name: *Platanus orientalis* L., Figure 2d,e). As shown in Figure 2f, the bark of plane trees exhibits continuous micro‐ridges and micro‐valleys, and various spherical micropores form in the micro‐ridges. Through the casting process, a PDMS layer with opposite continuous micro‐ridges and micro‐valleys was obtained (Figure 2g). The microspheres in the micro‐valleys (Figure 2h), in combination with the continuous micro‐ridges and micro‐valleys finally formed the bionic dual‐state amplifying microstructures. MXene is regarded as a promising sensing material for flexible stimuli‐responsive sensors due to its large specific surface area and outstanding metallic electrical conductivity.^[^ ^41^ ^]^ Hence, Ti_3_C_2_T_x_‐MXene, selected as the intermediate conductive layer, was spray‐coated on the micro‐structured PDMS layer. Ti_3_C_2_T_x_ was prepared by etching Al in the precursor Ti_3_AlC_2_ (MAX phase) with LiF and HCl.^[^ ^42^ ^]^ As shown in Figure 2i, energy‐dispersive X‐ray spectroscopy (EDS) element mapping illustrates the homogenous distribution of MXene over the micro‐structured PDMS layer, with the element content shown in Figure S2 (Supporting Information).

### 2.2 TPD Sensing Mechanism and Performance

**Figure 3:**
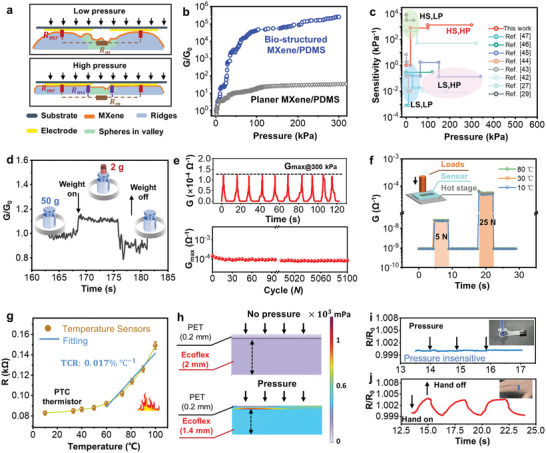
Sensing mechanism and performance of the TPD e‐skin. a) Working mechanism of the TPD pressure sensor with two‐stage enhancement and its equivalent circuit. b) Pressure sensitivities of the bio‐structured pressure sensor and pressure sensor without structure. c) Comparison of the sensitivity and range between the proposed TPD pressure sensor and conventional microstructure‐based pressure sensors. HS: high sensitivity. LS: low sensitivity. HP: high pressure. LP: low pressure. d) The ability of the TPD pressure sensor to detect a small pressure under the preload of 5 kPa, where *G*_0_ is the conductance of the preload pressure of 5 kPa. e) Cyclic testing of the TPD pressure sensor: time‐dependent performance of successive 10 cycles (top) and maximum conductivity change over 5000 cycles (bottom). f) Temperature insensitivity of the TPD pressure sensor. g) Response of the CB thermistor in the TPD e‐skin to temperatures. h) FEA simulation results show the deformation of two layers under pressure. i) Pressure insensitivity of the CB thermistor. j) The CB thermistor responds to hand temperature.

Due to the bionic contact interface with the dual‐state amplifying microstructure, the pressure sensor exhibits high‐pressure sensitivity over a wide range and temperature insensitivity. To characterize the electrical performance, a pressure of 0–300 kPa was applied to the TPD e‐skin. The resistance decreased continuously from the order of 10^9^ Ω to 10^3^ Ω (Figure S3, Supporting Information). To thoroughly investigate the piezoresistive response of the pressure sensor, an equivalent circuit diagram is presented as shown in **Figure** **3**a. The total resistance of the pressure sensor (*R*_Total_) is composed of the contact resistance of the MXene layer on the micro‐ridges and the Ag electrode (*R*_mr_), the contact resistance of the MXene layer on spheres in the micro‐valleys and the Ag electrode (*R*_ms_), and the film resistance of the MXene layer on the bionic structure between the two adjacent interdigitated electrodes (*R*_m_). Therefore, the total resistance of the pressure sensor can be expressed as

(1) $$R_{\mathrm{Total}}=R_{\mathrm{mr}}\;+R_{\mathrm{ms}}+R_{m}$$

By rewriting *R*_mr_, *R*_ms,_ and *R*_m_, replacing them with resistivity (*ρ*), length (*L*), the thickness of the MXene layer (*T*_m_), contact area (*A*_c_), and surface resistivity of the contact interface between the MXene layer and Ag electrode (*ρ*_ma_), the above equation can be given as

(2) $$R_{\mathrm{Total}}=\rho_{\mathrm{ma}}\;A_{\mathrm{cmr}}+\rho_{\mathrm{ma}}A_{\mathrm{cms}}+\rho L_{m}/T_{m}$$

In this equation, ρ and ρ_ma_ are treated as constants in order to simplify the analysis while not losing generality. Since monolayer MXene is adopted to form the conductive layer, *T*_m_ is also pressure independent and assumed constant.

As the applied pressure increases, the change in the total resistance of the pressure sensor compared to the initial state can be given as

(3) $$\begin{array}{ccc} \Delta R & = & R_{\mathrm{Total}}^{1}\;-\;R_{\mathrm{Total}}^{0}=\rho_{\mathrm{ma}}\;\left(A_{\mathrm{cmr}}^{1}-A_{\mathrm{cmr}}^{0}\right)+\rho_{\mathrm{ma}}\left(A_{\mathrm{cms}}^{1}-A_{\mathrm{cms}}^{0}\right)\\ & & +\;\rho \left(L_{m}^{1}-L_{m}^{0}\right)/T_{m} \end{array}$$

When the applied pressure is low, the MXene layer on microspheres in the micro‐valleys cannot form contact with Ag electrodes. Besides, in this stage, the order magnitude of *R*_m_ (10^2^) is far smaller than that of *R*_mr_ (10^5^–10^9^), and the influence of the decrease of length (*L*_m_) on the resistance can be neglected. In this stage, Equation (3) can be simplified as

(4) $$\Delta R\;=R_{\mathrm{Total}}^{1}\;-\;R_{\mathrm{Total}}^{0}=\rho_{\mathrm{ma}}\;\left(A_{\mathrm{cmr}}^{1}-A_{\mathrm{cmr}}^{0}\right)$$

Therefore, in the first stage, relatively low pressure induces contact between the MXene on the micro‐ridges. Therefore, the increase in contact area as the pressure increases subsequently leads to a decrease in resistance.

As the pressure is further increased, the MXene layer on spheres in the mico‐valleys will come into contact, thereby creating a richer variation in the contact area. In this stage, the contact effect between the MXene layer on the micro‐ridges disappears. Besides, *R*_mr_+*R*_ms_ (10^3^–10^4^) is about two orders of magnitude larger than *R*_m_. Hence, the influence of *R*_m_ could still be neglected. In this stage, Equation (4) can be simplified as

(5) $$\Delta R\;=R_{\mathrm{Total}}^{2}\;-\;R_{\mathrm{Total}}^{1}=\rho_{\mathrm{ma}}\;A_{\mathrm{cmr}}$$

Therefore, in the second state, the spheres in the micro‐valleys play a significant role in the resistance change. The sudden increase in the contact area due to the small size of MXene‐coated spheres (diameter < 20 µm) furtherly brings light to the decrease of the whole resistance. The compression deformation of the pressure sensor during the pressure load keeps increasing (Figure S4, Supporting Information). These bionic dual‐state microstructures prevent premature saturation via the formation of the relay contact effect between the MXene‐coating sphere layer and Ag electrodes, hence remarkably improving the pressure‐sensing performance.

Figure 3b shows the electrical performance of the pressure sensor as a function of applied pressure. The pressure sensitivity is denoted as δ(△*G*/*G*_0_)/δ(P).^[^ ^38^ ^]^ The pressure sensor maintains a sensitivity of 795 kPa^−1^ (50–100 kPa). Besides, owing to the bionic dual‐state amplifying microstructure, the resistance of the pressure sensor continuously decreases even at ultra‐high‐pressure loads (Figure S5, Supporting Information). As shown in Figure 3b, the pressure sensor exhibits a higher linear sensitivity of 1316 kPa^−1^ over a wide range (100–300 kPa). Figure S6 (Supporting Information) shows the detailed pressure sensitivity and pressure range. As shown in Figure 3b and Figure S7 (Supporting Information), the pressure sensor with bionic dual‐state amplifying microstructure from the bark of plane trees exhibits high sensitivity under the applied pressure at a range from 0 to 300 kPa than that without structure. Especially, the sensitivity of our pressure sensor is improved by six orders of magnitude than that without structure. The superiority of the pressure sensor that exhibits high sensitivity over a wide pressure range is furtherly confirmed through comparison with other conventional microstructure‐based pressure sensors (Figure 3c).^[^ ^28^, ^30^, ^43^, ^44^, ^45^, ^46^, ^47^, ^48^ ^]^ The pressure sensor is responsive to small pressure even under a relatively high preload of 5 kPa. The pressure sensor could detect the addition of a little weight of ≈19.6 mN (Figure 3d). The cyclic loads were applied to the pressure sensor and the maximum conductance *G*_max_ (*G* = 1/R) at 300 kPa was recorded to investigate the durability of the sensor. The pressure sensor shows reversible behavior and stable *G*_max_ over the first 10 cycles (top inset in Figure 3e). Due to the anchor and protection of Ecoflex layer, the pressure sensor shows excellent mechanical stability. After 5000 cyclic loadings, the *G*_max_ of the sensor remains stable over the next 100 cycles (bottom inset in Figure 3e), and the maximum resistance deviation at 300 kPa over 5000 cycles is within 8.1% (Figure S8, Supporting Information). The different samples exhibit nearly identical pressure sensing performance, with very little variance between devices (Figure S9, Supporting Information).

The TPD e‐skin was then placed on a hot plate to test the influence of temperature on the pressure response. As shown in Equation (1), under a low‐pressure load, the order magnitude of *R*_mr_ +*R*_ms_ is much larger than that of the R_m_, the resistance the pressure response of the contact resistance sensor is mainly determined by the resistance of the contact interface formed by the conductive functional material and the electrodes, the pressure sensor shows negligible resistance changes to the different ambient temperatures. When under a high‐pressure load, the order magnitude of *R*_m_ is close to that of *R*_mr_+*R*_ms_, so the change in resistance of R_m_ induced by temperature could affect *R*_Total_. Yet, due to the inherently temperature‐insensitive contact resistance modulation, the pressure sensor only shows a 5.6% resistance decrease for a 70 °C increase in temperature (Figure 3f), which reduces the temperature response by 91.2% compared to that of MXene film (Figure S10, Supporting Information).

The positive temperature coefficient (PTC) thermistor was fabricated by screen‐printed CBconductive ink on the PET film. As the temperature increases, the CB compound expands, the distance between the CBs increases, and the resistance of the thermistors increases. Figure 3g illustrates the responses of the CB thermistor to the environment temperatures with a temperature coefficient of resistance (TCR), denoted as Δ*R*/(*R*_0_Δ*T*), of 0.017% °C^−1^ over a temperature range between 60 and 100 °C, demonstrating its capability of detecting temperature changes.

Due to the deformation caused by the applied pressure, the CB film thermistor could generate cracks. As a result, the resistance of the CB sensor will increase, which could reduce the accuracy of temperature monitoring. To eliminate the influence of pressure on temperature sensing, a soft, low‐modulus Ecoflex was placed under the pressure sensor. In this high‐low modulus configuration inspired by human skin, the energy from the applied pressure could be dissipated by Ecoflex. As shown in Figure 3h, The finite element analysis (FEA) simulation results show that the Ecoflex is compressed under pressure but the PET film does not deform. As a result, the CB thermistor exhibits negligible resistance changes to the external pressure (Figure 3i). Besides, the CB thermistor responds to hand temperature (Figure 3j), exhibiting good cyclic stability.

### 2.3 Configuration and Sensing Performance of Wireless Prosthetic E‐skin

A wireless circuit system for signal acquisition and processing was designed (Figure S11, Supporting Information), with the photograph shown in Figure S12 (Supporting Information). The TPD e‐skin is connected with the signal processing module via soft Dupont lines to avoid possible mechanical and electrical failure caused by the elastic modulus mismatch between the TPD e‐skin and the rigid signal processing module. Each signal could be pre‐processed by the signal conditioning circuits and digitized by an analog‐to‐digital converter (ADC) in the microcontroller unit (MCU). The digital signals are then further processed and sent wirelessly to another terminal (i.e., personal computers) through Bluetooth low energy (BLE).

Since the temperature and pressure sensors detect external stimuli through resistance changes, the voltage divider circuit (Figure S13, Supporting Information) was chosen as the main part of the signal conditioning circuit, which converts the value of the resistance change into a voltage according to Ohm'slaw. The constant resistor in the voltage divider circuit is rationally selected in accordance with the range of the output resistance under the external pressure/temperature. In this TPD e‐skin system, the constant resistor value in the temperature divider circuit was set similarly to that of the temperature sensor at room temperature (≈10 °C), with the voltage/temperature relationship shown in Figure S14 (Supporting Information). By contrast, as the initial resistance of the pressure sensor is very larger (≈10^9^ Ω) and is decreased monotonically to ≈10^3^ Ω as the external pressure increased, the constant resistor value in the voltage divider circuit was set at ≈1.2 × 10^5^ Ω, with the voltage/pressure relationship shown in Figure S15 (Supporting Information). The pressure sensor responds to the pressure quickly, with a fast response time of 18 ms under a pressure of ≈45 kPa (Figure S16, Supporting Information).

We then fixed the TPD e‐skin to a mouse and investigated its dual response to a mouse click, double click, and drag action (Figure S17, Supporting Information). Concerning the mouse click and double click, distinct peaks can be clearly observed in both temperature and pressure signals during each click. Then, the TPD e‐skin was also fixed on a robotic arm to investigate its dual response to different objects (Figure S18, Supporting Information), capable of simultaneously detecting the applied pressure and thermal properties of contact objects (e.g., a glass and a hot bottle with a temperature of 60 °C). In addition, the TPD e‐skin system was attached to a robotic gripper to further demonstrate its capability to understand tactile information in various manipulation actions (Figure S19, Supporting Information). No obvious signal fluctuations when the robotic gripper is not operating (*t*_1_). When the robotic gripper starts to grip the bottle firmly (*t*_2_), the electrical output of the pressure sensor in the TPD e‐skin increases sharply. When hot water is poured into the bottle (*t*_3_), the temperature sensor in the TPD e‐skin responds to the temperature change, but the electrical output of the pressure sensor does not fluctuate, demonstrating its temperature insensitivity. Both temperature and pressure responses remain stable throughout the holding process. The final operation is to release the robot gripper to separate the gripper and bottle (*t*_4_). Accordingly, the dual electrical output of the TPD e‐skin is restored to its original state.

### 2.4 Neural Coding for Temperature‐Pressure Signals

Frequency modulation for electrical signals could interface with biological nervous systems, promoting a sense of ownership for amputees, or a sense of embodiment in scenarios of remote control in discontinuous space via virtual reality.

**Figure 4:**
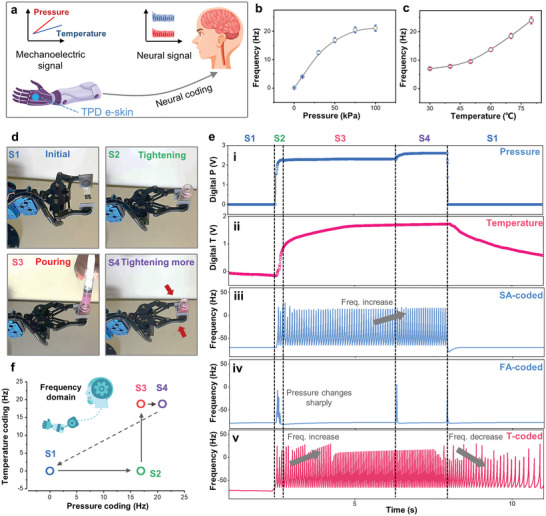
Frequency modulation for the TPD e‐skin system. a) Schematic showing the neural coding of TPD e‐skin for prosthetics and remote control. b,c) Frequency response as a function of pressure and temperature. d) Illustrations of the four kinds of consecutive operations. e) The corresponding dual electrical response and neural‐coded frequency signals for the tactile motion via the TPD e‐skin system. i) Pressure response during four kinds of consecutive operations. ii) Temperature response during four kinds of consecutive operations. iii) SA‐coded spikes for the static component in the pressure signal. iv) FA‐coded spikes for the dynamic component in the pressure signal. v) T‐coded spikes for the temperature signal. f) Four operational states in the frequency domain with easily distinguishable features.

First, mimicking the functions of SA and FA receptors, and thermoreceptors, a mathematical (Izhikevich) neuron model was implemented through computation in Python to simultaneously and independently encode the dynamic and static components of pressure and temperature signals, respectively, into frequency signals (**Figure** **4**a).^[^ ^49^, ^50^ ^]^

The neural model for static components in pressure signals and temperature signals is shown in Note S1 (Supporting Information). Through this model and computational methods within a Python‐based program, the static pressure and temperature information are converted into nerve‐acceptable spikes, with the coded frequency of the spikes increasing with pressure and temperature (Figure 4b,c). In addition, the Izhikevich‐based customized neuron model was also implemented to neurally code the dynamic components in pressure signals into frequency signals (Note S2, Supporting Information).

Then, an interesting example of the encoding of dual tactile information into nerve‐acceptable frequency signals in various manipulation actions of robotic hands is demonstrated, with the manipulation actions shown in Figure 4d. The dual electrical output of the TPD e‐skin responding to the four states (S1: no operation; S2: grasping the bottle; S3: pouring water; S4: grasping the bottle with a larger force) is depicted in Figure 4d. The electrical output of the temperature sensor does not fluctuate when the gripping force increases in state 4, demonstrating its insensitivity to pressure. Figure 4e shows the SA‐, FA‐, and temperature‐coded neural spikes. As seen in Figure 4eiii, the frequency of the SA‐coded spike increases with the grasping force (S1, S3, and S4). The FA‐coded spike is generated only when the grasping force changes sharply (Figure 4eiv, S1→S2, S3→S4, S4→S1). In addition, the frequency of the temperature (T)‐coded spike increases as hot water is poured into the bottle (Figure 4ev, S2→S3). Figure 4f shows that the four states are also distinguished in the frequency domain, proving that these frequency spikes are suitable for the biological nerve center to learn valuable cues.

### 2.5 Machine‐Learning‐Assisted Analysis of Frequency Signals for Object Cognition

**Figure 5:**
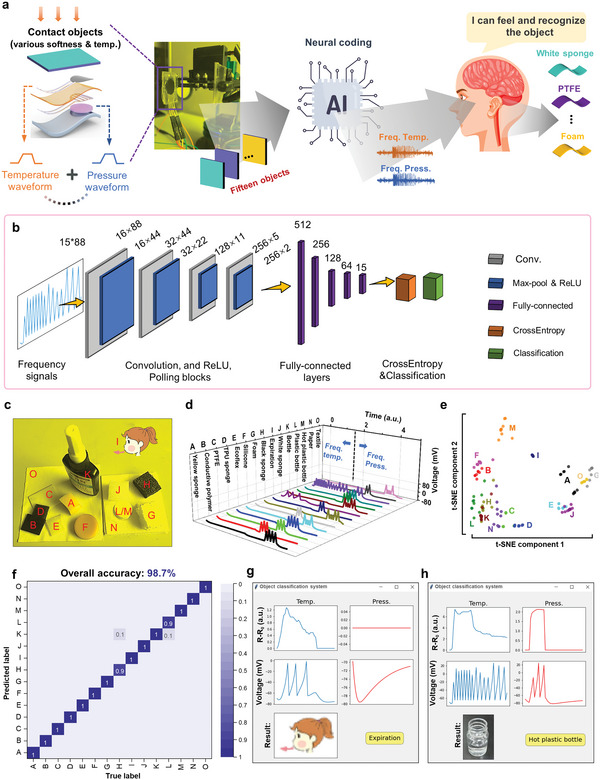
Demonstration of brain‐like object recognition system based on neurally coded frequency signals. a) Machine‐learning‐assisted object recognition process, including signal generation and acquisition, frequency modulation, data analysis, and object cognition. b) Detailed framework of the constructed 1D‐CNN model. c) Photograph of the 15 objects. d) Temperature‐pressure frequency waveforms generated by 15 kinds of objects, which are neurally coded. e) Visualization of temperature‐pressure frequency data within 150 samples via t‐SNE dimensionality reduction. f) Confusion matrix of object recognition. g) Display of the cognition result of expiration and its corresponding waveforms. h) Display of the cognition result of the hot plastic bottle, and its corresponding waveforms.

The above demonstrations successfully show that by emulating the skin structure and adopting the neural model, the TPD e‐skin is capable of independently sensing pressure and temperature, and encoding them into neurally acceptable frequency signals, which are capable of interfacing with human biological systems. It is well known that the human system independently obtains thermal and pressure neural signals through thermoreceptors and mechanoreceptors, respectively. Then, the two thermal and pressure signals are analyzed in the brain for object recognition or situation understanding. Here, we first independently coded the neural signals of thermal and pressure through two copies, implemented using Python programming language, of the neural model, and then adopt a machine learning algorithm to analyze the two signals together for object recognition (**Figure** **5**a,b). As shown in Figure 5a, the TPD e‐skin was attached to the robotic hand to grasp 15 different objects/motions (yellow sponge, conductive polymer, poly(tetrafluoroethylene) (PTFE), thermoplastic polyurethane (TPU) sponge, Ecoflex, foam, black sponge, exhalation, white sponge, bottle, plastic bottle, hot plastic bottle, paper, and textile) (Figure 5c) at a constant speed and maximum pressure of 5 N to obtain the bimodal pressure and temperature information. Then, these bimodal electronic signals are neurally coded into nerve‐acceptable frequency signals and analyzed by a brain‐like artificial one‐dimensional convolutional neural network (1D CNN) (Figure 5b) for object recognition.

For each object, two pressure and temperature signals, corresponding to the softness and thermal properties, were recorded during the grasping process. For each object, the temperature and pressure signals were recorded with 44 points in 2 s. In the later process, the two signals were concatenated into one signal with a dimension of 88. The frequency waveforms of the extracted 15 materials are shown in Figure 5d. As can be seen, the pressure and temperature frequency signals vary significantly between the different materials. The total sample size for 15 objects is 1500. The t‐distributed stochastic neighbor embedding (t‐SEN) was then used to visualize the group of 150 frequency signals (10 samples for each object, Figure 5e). Each point represents the temperature‐pressure information of an object projected from 1 × 88 data in 2 dimensions. Points of the same object category (i.e., same color) are clustered together, forming ≈15 categories (A‐O). These results show that frequency data can provide valuable clues for object recognition in the frequency domain. To account for device variation and hysteresis, the raw frequency data was first normalized before being used for object recognition. Then, 70% of the samples within the data set were used for training, 10% for validation, and 20% for testing. The detailed brain‐like object recognition process of the system is shown in Figure 5b. The 1D CNN model is constructed from four convolutional layers and five fully connected layers. The convolutional layers learn a temporal representation of the temperature‐pressure information in a low‐cost and energy‐efficient manner. After training, the final recognition performance reached 98.7% (Figure 5f). An example of the results for the exhalation and hot plastic bottle is shown in Figure 5g,h, where the corresponding temperature and frequency modulated waveforms (blue line), pressure waveforms and frequency modulated waveforms (red line), and the recognition results after grasping are shown. This demonstration proves that the TPD e‐skin system is expected to provide new tactile sensation and object recognition capabilities for advanced prosthetics and expand the development of artificial intelligence.

## 3 Conclusion

In this work, we developed a TPD e‐skin system with decoupled temperature‐pressure sensing and nerve‐acceptable neural coding capability. The TPD e‐skin features a bionic skin‐like hierarchical structure and high‐low modulus configuration, which could be rapidly and inexpensively fabricated by screen‐printing technique and layer‐by‐layer assembly. Benefiting from the relay effect of the bionic dual‐state amplifying microstructure cast from the bark of plane trees, the TPD e‐skin exhibits a high sensitivity (795 kPa^−1^ at 30–50 kPa and 1319 kPa^−1^ at 100–300 kPa) and a broad pressure‐sensing range over 300 kPa. The pressure sensor in the TPD e‐skin relies on contact resistance modulation via external pressure. This contact resistance is not inherently sensitive to temperature and could reduce the temperature response by 91.2% compared to that of pure MXene film. In addition, the high‐low modulus configuration of PET‐Ecoflex film makes the temperature in the TPD e‐skin pressure insensitive. Moreover, the TPD e‐skin system with a customized signal processing and wireless transmission module, which does not require bulky measuring instruments, is appropriate for practical versatile robotic and prosthetics applications. Finally, through computing neural modal, the bimodal temperature‐pressure signals are encoded into three‐type frequency signals, similar to SA receptors, FA receptors, and thermoreceptors. Four operational states in the time domain are also distinguished in the frequency domain after neural coding. Moreover, a brain‐like fusion process based on 1D CNN for neurally coded frequency signals is developed to recognize 15 kinds of objects with a high accuracy of 98.7%. Our work provides a new route toward advanced intelligent prosthetic skin to interface with biological systems in a nerve‐acceptable manner. In future work, we will focus on the optimization of the TPD e‐skin neural system in the aspect of sensor, hard system, and algorithms, and strive to transplant the system into a more miniaturized system, so as to possess a higher application value.

## 4 Experimental Section

### Preparation of Ti_3_CT_x_‐MXene

Powders (1.0 g) of Ti_3_AlC_2_ (400 mesh, Jilin 11 technology) were added to 20 mL of 6 m HCl (Aladdin) solution with 10 g LiF (Aladdin) to selectively etch the Al from the Ti_3_AlC_2_ MAX raw powders. The etching reaction lasted for 35 h under stirring (500 rpm) at a constant temperature of 35 °C. The suspension was then centrifuged at 6000 rpm for 10 min, followed by washing with deionized water several times until the pH of the dispersion was ≈6. The multilayered Ti_3_C_2_T_x_ powders were obtained, which were then dispersed in 50 mL of deionized water and delaminated by ultrasonication for 1 h. Then, unexploited multilayered Ti_3_C_2_T_x_ powders were removed by centrifugation at 4000 rpm for 5 min. After drying for ≈15 min at 60 °C, few‐layered Ti_3_C_2_T_x_ powders were obtained. Next, the obtained few‐layered Ti_3_C_2_T_x_ powders of 0.5 g were added to 50 mL deionized water, which was stirred (400 rpm) for 30 min at 50 °C, followed by ultrasonication for 10 min to prepare 5 mg mL^−1^ MXene aqueous dispersion.

### Preparation of MXene‐Coated PDMS with the Dual‐State Amplifying Microstructures

The bark of plane trees (from Southeast University, China) was cut into rectangles (10 mm × 10 mm) and washed two times with ethanol absolute (Aladdin, China) and two times with deionized water (Aladdin, China). After being dried, the bark of plane trees was fixed to a glass substrate with double‐sided tapes. Polydimethylsiloxane (PDMS, SYLGARD 184 Silicone Elastomer, China) prepolymer was prepared with a base‐to‐curing agent ratio of 10:1 and degassed in a vacuum oven for 30 min to remove air bubbles. Then, the PDMS mixture was spin‐coated (200 rpm) onto the bark. After curing at 80 °C for 30 min, PDMS film with opposite structures of the bark was peeled off. The 5 mg mL^−1^ MXene aqueous was sprayed on the PDMS surface, forming the uniform conductive pressure‐sensing layer.

### Preparation of 3D‐Integrated TPD E‐Skin

First, polyethylene terephthalate (PET) film was cut into rectangles (35 mm × 75 mm) and washed with ethanol absolute. The circular thermistor was fabricated by screen‐printing the carbon black (CB) ink (weight ratio of CB ≈25%, Shenzhen JieYongCheng Electronics Co., Ltd, China) on the PET film and dried at 80 °C for 30 min. Then, two parallel Ag electrodes were fabricated by screen‐printing the Ag ink (Shenzhen JieYongCheng Electronics Co., Ltd, China) on the PET film as electric leads of the CB thermistor, which dried at 80 °C for 30 min. Next, the Ag interdigitated electrode was screen‐printed on the other side of the PET film and dried at 80 °C for 30 min. Then, the MXene‐coated micro‐structured PDMS adhered to the PET film with the Ag interdigitated electrode using the water‐based acrylic pressure‐sensitive adhesives (808, Wending Sticky Treasure, China). Then, Ecoflex (0030) prepolymer was prepared by mixing part A to part B ratio of 1:1 by weight, which was then poured onto the PET film with microstructured PDMS to form the buffer after being dried at 80 °C for 25 min. Finally, conductively connecting devices were fixed on the Ag electrodes to connect with soft Dupont lines.

### Characterizations

A field‐emission scanning electron microscope (SEM, Quanta 200 FEI) was used for characterizing the morphology of the bark of plane trees and MXene‐coated micro‐structured PDMS. Electrical characterization of TPD e‐skin was performed using a source meter (Keithley 2400). A force gauge (Pubtester, TST‐01H) was used to apply pressure and compress the TPD e‐skin.

## Conflict of Interest

The authors declare no conflict of interest.

## Supporting information

Supporting Information

## Data Availability

The data that support the findings of this study are available from the corresponding author upon reasonable request.
