# Supplementary material for: Bioinspired Young's Modulus‐Hierarchical E‐Skin with Decoupling Multimodality and Neuromorphic Encoding Outputs to Biosystems
Source: Adv Sci (Weinh). 2023 Sep 7;10(31):2304121. doi: 10.1002/advs.202304121 (PMC10625104; doi:10.1002/advs.202304121)
Supplement: Supplementary file 1 — Supporting Information [file ADVS-10-2304121-s001.pdf]

ADVANCED SCIENCE
Open Access

Supporting Information

for *Adv. Sci.*, DOI 10.1002/advs.202304121

Bioinspired Young's Modulus-Hierarchical E-Skin with Decoupling Multimodality and Neuromorphic Encoding Outputs to Biosystems

Shengshun Duan, Xiao Wei, Fangzhi Zhao, Huiying Yang, Ye Wang, Pinzhen Chen, Jianlong Hong, Shengxin Xiang, Minzhou Luo, Qiongfeng Shi, Guozhen Shen* and Jun Wu**

Bioinspired Young's Modulus-Hierarchical E-Skin with Decoupling Multimodality and Neuromorphic Encoding Outputs to Biosystems

Shengshun Duan, Xiao Wei, Fangzhi Zhao, Huiying Yang, Ye Wang, Pinzhen Chen, Jianlong Hong, Shengxin Xiang, Minzhou Luo, Qiongfeng Shi, Guozhen Shen*, and Jun Wu**

S. Duan, X. Wei, F. Zhao, H. Yang, Y. Wang, P. Chen, J. Hong, S. Xiang, Q. Shi, J. Wu
Joint International Research Laboratory of Information Display and Visualization, School of Electronic Science and Engineering, Southeast University, Nanjing, 210096, China
Emial: qiongfeng@seu.edu.cn, wujunseu@seu.edu.cn
G. Shen
School of Integrated Circuits and ElectronicsBeijing Institute of TechnologyBeijing 100081, China
E-mail: gzshen@bit.edu.cn
M. Luo
Jiangsu Jitri Intelligent Manufacturing Technology Institute Co, Ltd, Photoelectric technology park of Jiangbei New District, Nanjing, 211500, China

Note S1. The neural model for static components in pressure signals and temperature. The dynamics of the member potential, *v*, corresponding to SA receptors and thermoreceptors in Izhikevich neuron modal is expressed:

$$\begin{cases} \frac{dv}{dt} = 0.04v^2 + 5v + 140 - u + kI \\ \frac{dv}{dt} = a(bv - u) \\ if\ (v \geq 30\ mV), then \begin{cases} v \leftarrow c \\ u \leftarrow u + d \end{cases} \end{cases}$$

Where a=0.02, b=0.2, c=-65 mV, d=2, k=0.75. In the above equation, u is the membrane recovery variable. I is the input current, calculated by V/R. a defines the characteristic time of recovery variable, and b defines the sensitivity of recovery variable. If the membrane potential reached the threshold value (30 mV), one spike was generated, and the membrane voltage and the recovery variable are reset. c and d contribute as well to defining the adaptation properties of the neuron.

Note S2. The neural model for dynamic components in pressure signals.

The dynamics of the member potential, v, corresponding to FA receptors in modified Izhikevich neuron modal is expressed:

$$\begin{cases} \dfrac{dv}{dt} = 0.04v^2 + 5v + 140 - u + \dfrac{kdI}{dt} \\ \dfrac{dv}{dt} = a(bv - u) \\ if\ (v\ \geq 30\, mV), then \begin{cases} v \leftarrow c \\ u \leftarrow u + d \end{cases} \end{cases}$$

Where a=0.02, b=0.2, c=-50 mV, d=2, k=5. Different form the original Izhikevich neuron modal, the value of δI/δt, other than I matters, which relates to rapid pressure changes within a short time frame. In the above equation, u is the membrane recovery variable. I is the input current, calculated by V/R. a defines the characteristic time of recovery variable, and b defines the sensitivity of recovery variable. If the membrane potential reached the threshold value (30 mV), one spike was generated, and the membrane voltage and the recovery variable are reset. c and d contribute as well to defining the adaptation properties of the neuron.

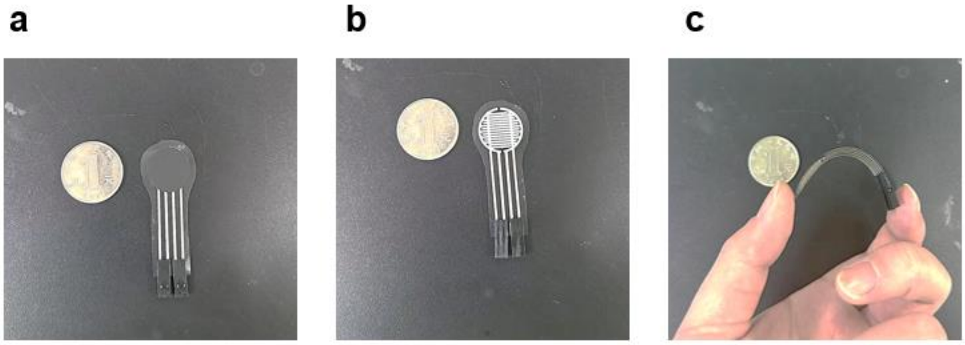

Figure S1. The screen-printed Ag coinductive and CB thermistor patterns on the PET film. **a.** The CB thermistor with two Ag conductive leads. **b.** The Ag interdigitated electrode for pressure sensors was screen-printed on the other side of the PET film. **c.** The PET film with functional patterns is bendable.

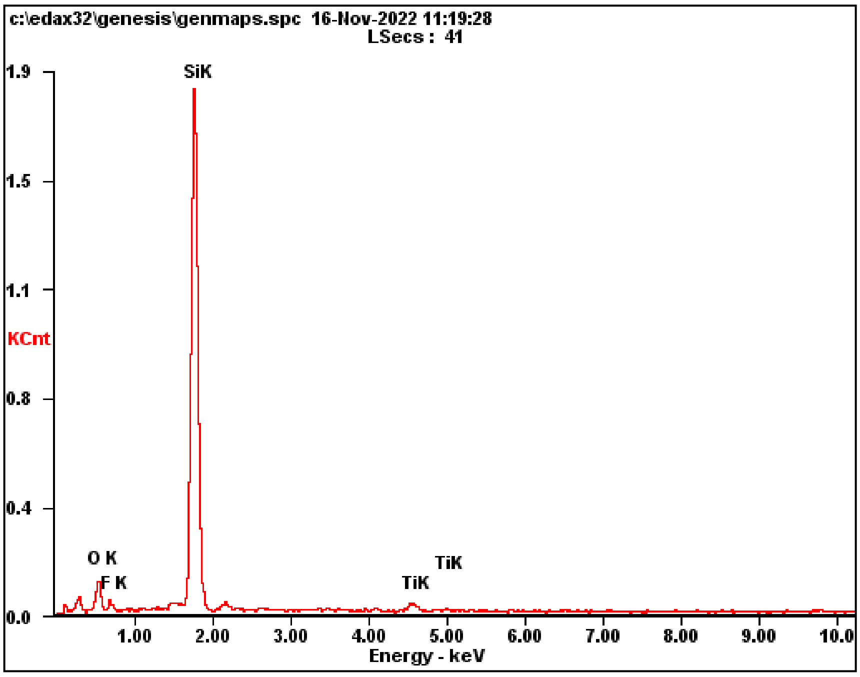

Figure S2. The element content in the Mxene-coated PDMS microstructures.

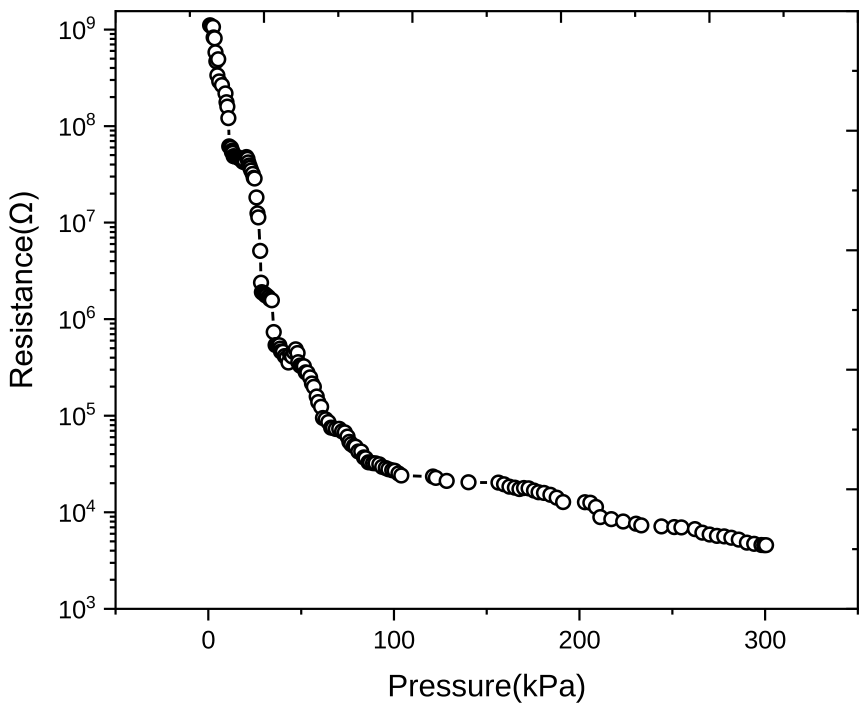

Figure S3. The resistance decreases continuously from the order of 10^9 Ω to 10^3 Ω as the applied pressure increases from 0 to 300 kPa.

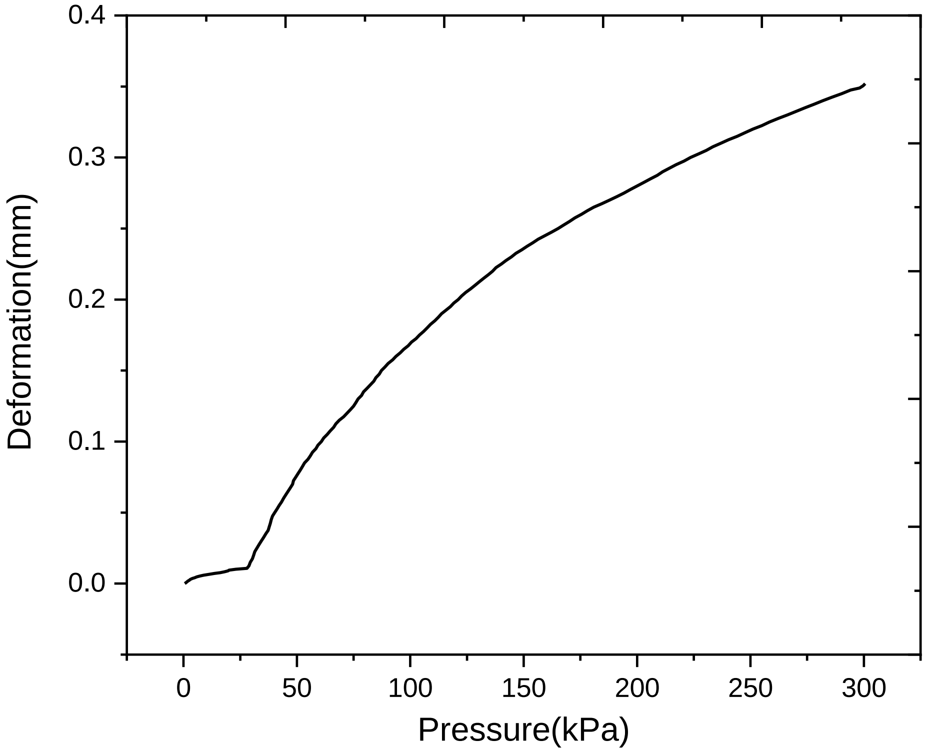

Figure S4. The compression deformation of the pressure sensor as the pressure load keeps increasing

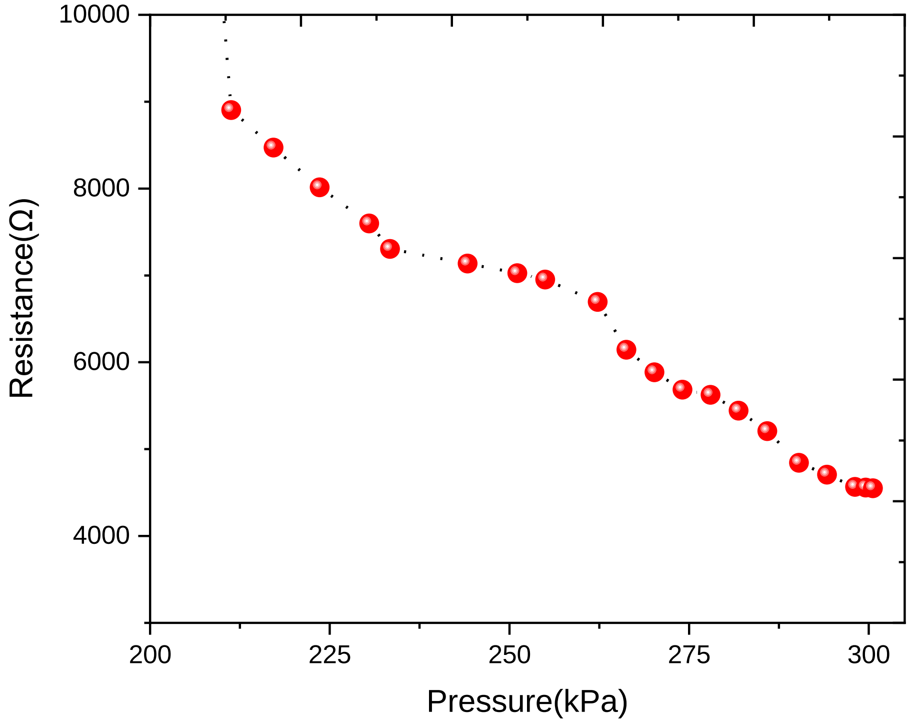

Figure S5. The resistance of the pressure sensor continuously decreases even at ultra-high-pressure loads over 200 kPa.

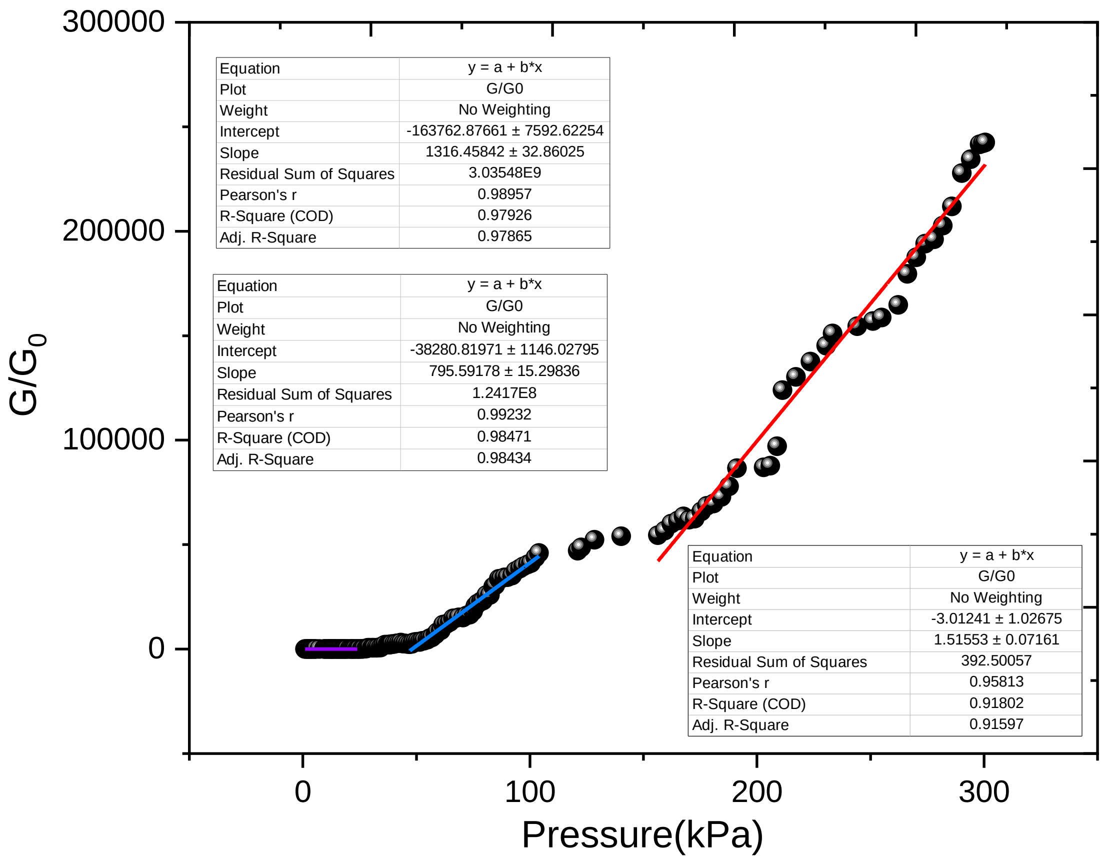

Figure S6. The detailed pressure sensitivity and related parameters of the pressure sensor at three pressure range.

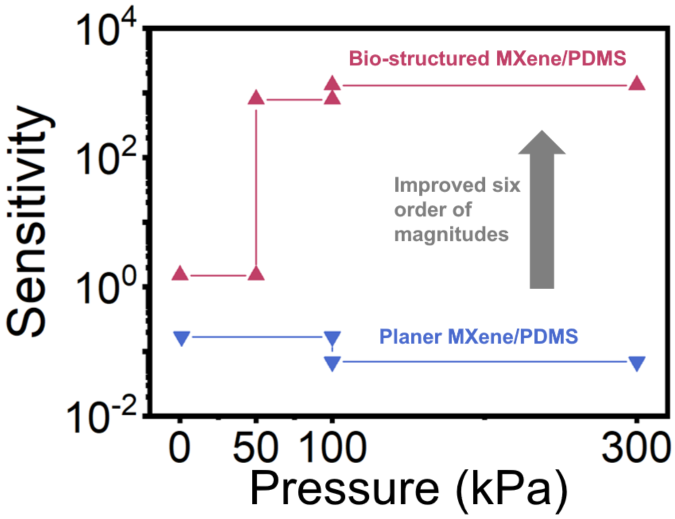

Figure S7. Sensitivity comparison of pressure sensors with bio-structures and pressure sensors planer structures.

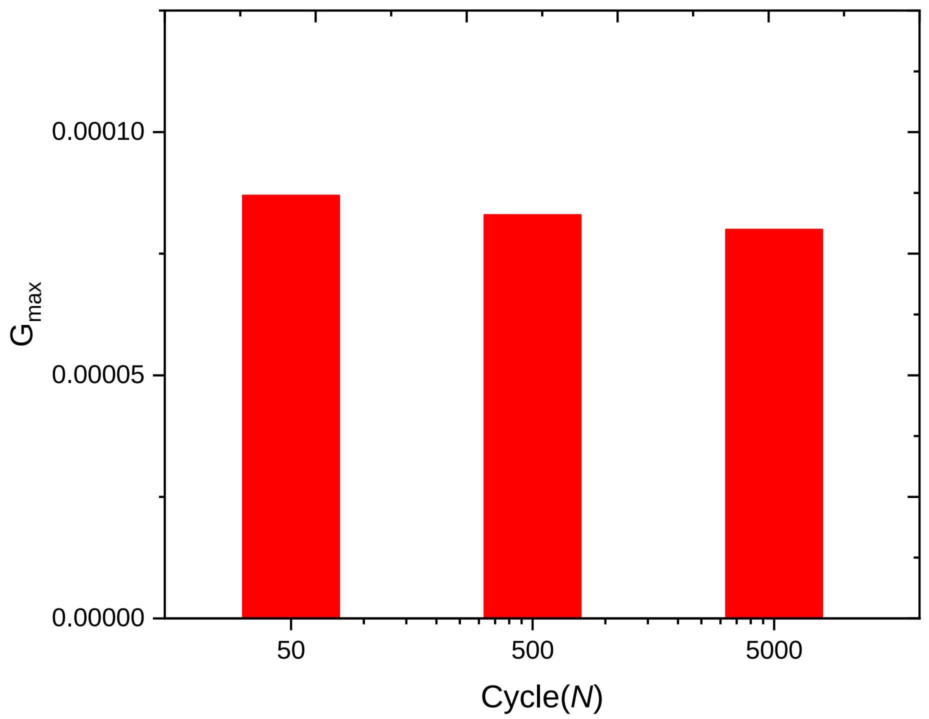

Figure S8. The conductance of the pressure sensor remained after 50, 500, and 5000 cycles of compressing cycles under the pressure of 300 kPa.

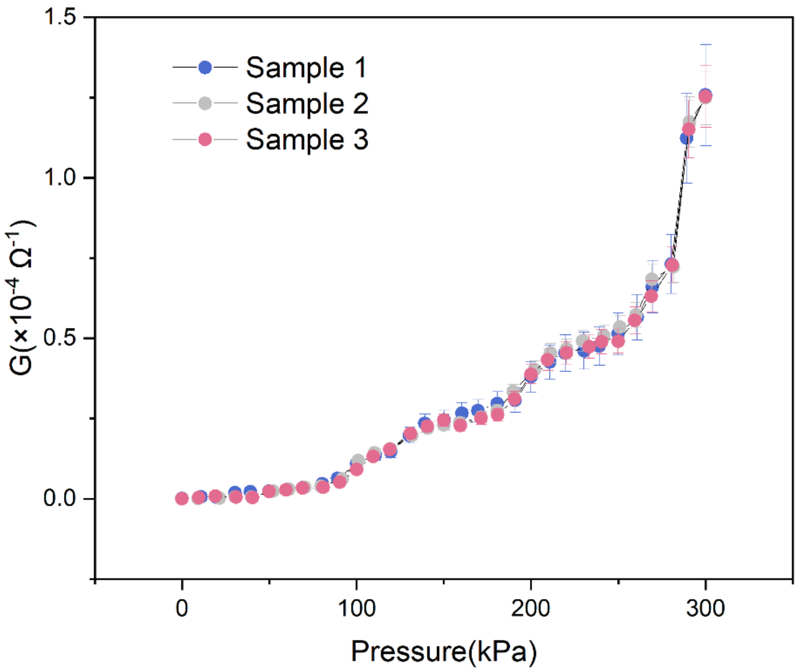

Figure S9. Pressure sensing curves of three different samples, exhibiting a little variance

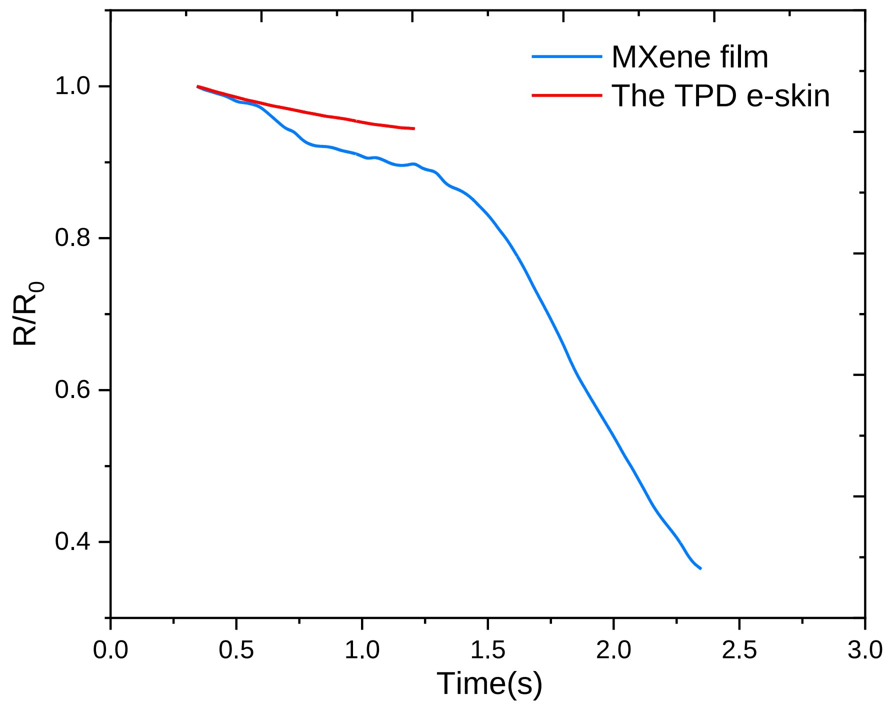

Figure S10. The resistance of the Mxene film and the pressure sensor in the TPD e-skin both changes as the temperature increases from 10 to 80 ℃. The resistance change of the pressure sensor reduces the temperature response by 91.2% compared to that of MXene film.

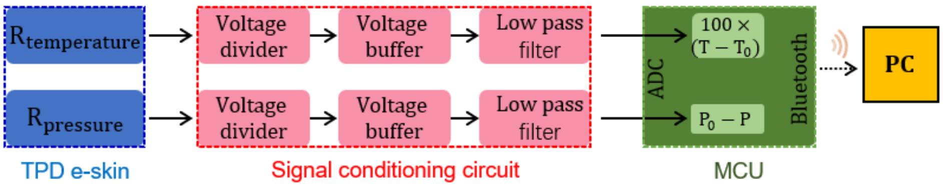

Figure S11. The block diagram of the TPD e-skin system including bimodal TPD e-skin, MCU, and BLE.

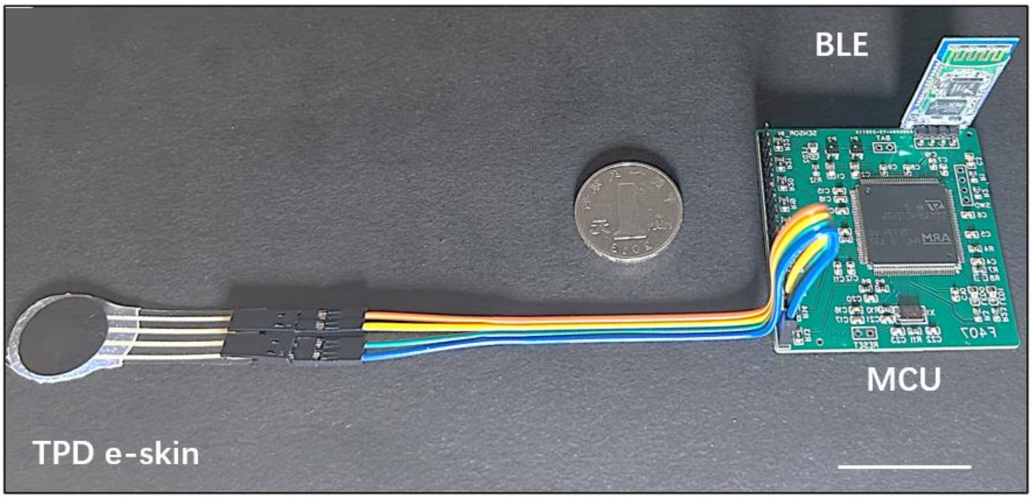

Figure S12. Photograph of the TPD e-skin system. Scale bar: 5 cm.

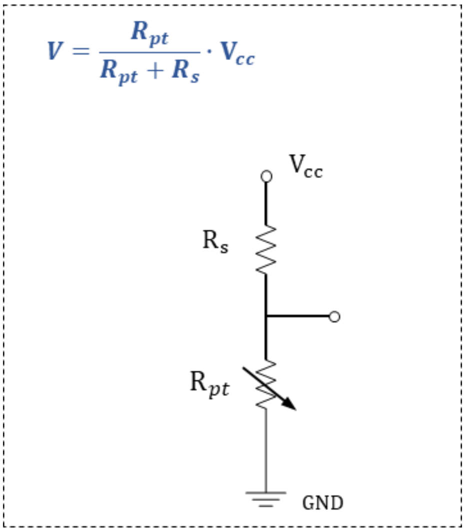

Figure S13. The voltage divider circuit.

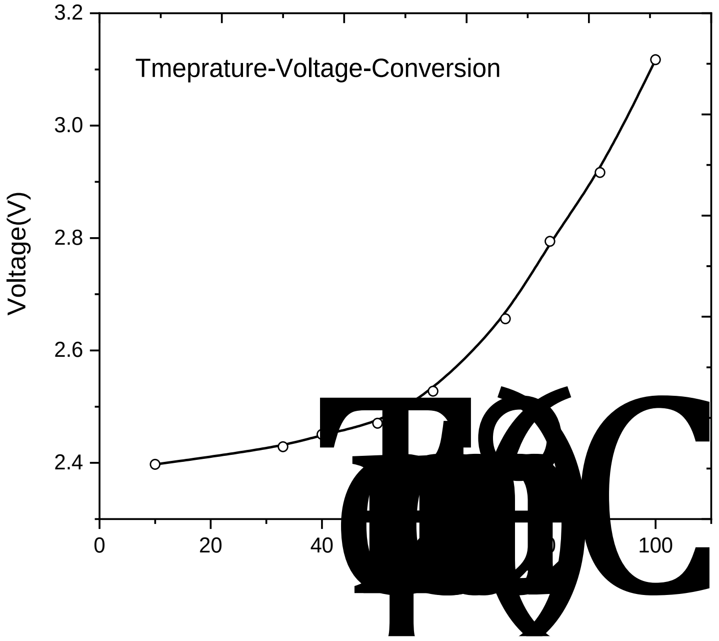

Figure S14. The volate/temperature relationship via the divider circuit.

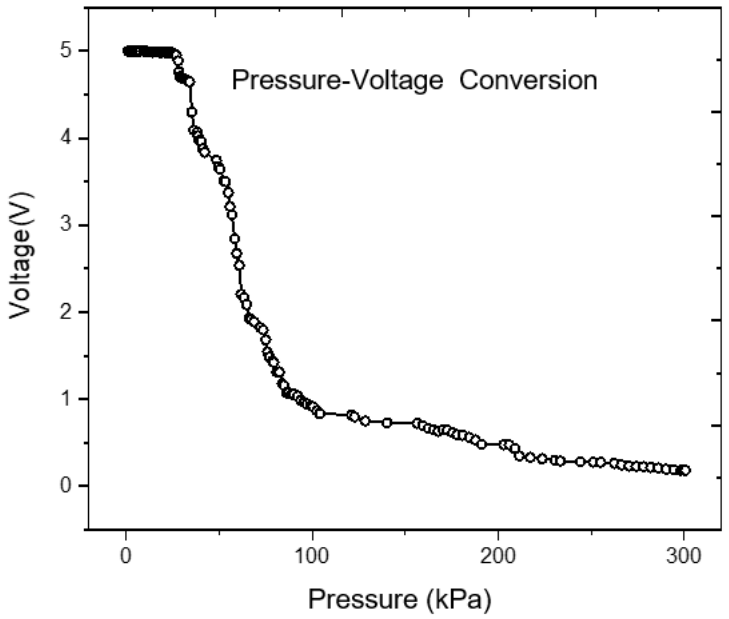

Figure S15. The volate/pressure relationship via the divider circuit.

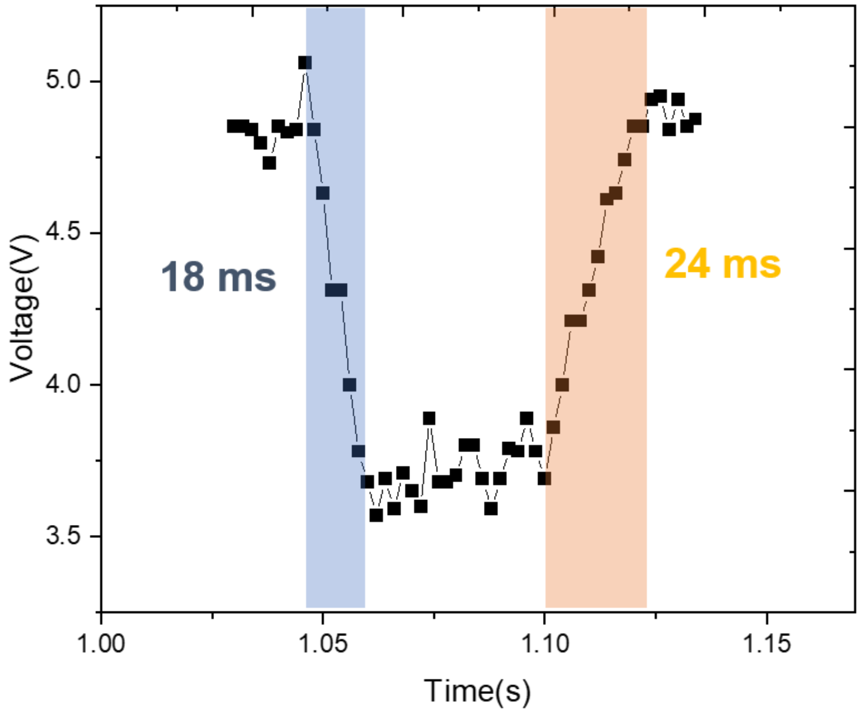

Figure S16. The pressure sensor shows a response time of 18 ms and a recovery time of 24 ms under a pressure of ~ 45 kPa.

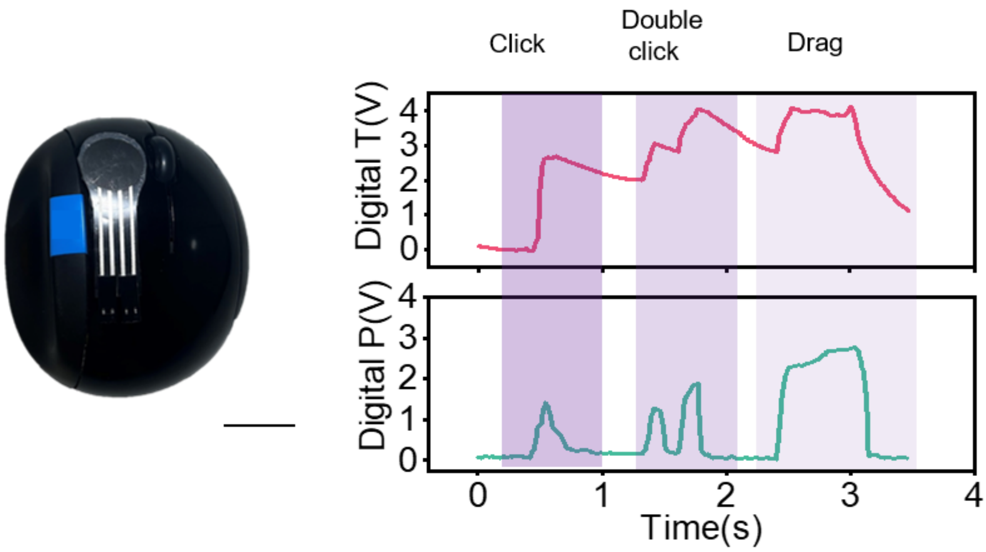

Figure S17. Photograph showing that the TPD e-skin was fixed on a mouse. Scale bar: 2 cm. The dual electrical response of the TPD e-skin for mouse operation of click, double click, and dragging.

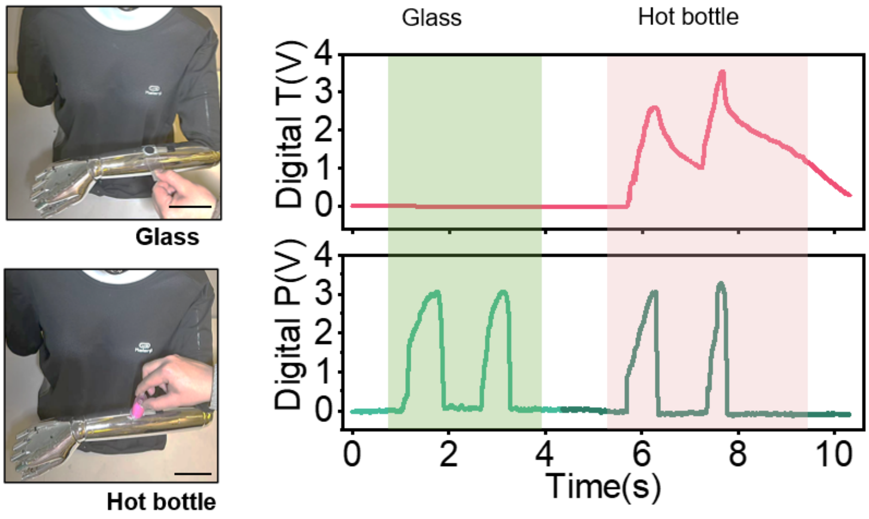

Figure S18. Photographs showing that the TPD e-skin was fixed on a robotic arm. Scale bar: 10 cm. The dual electrical response of the TPD e-skin for different contact objects.

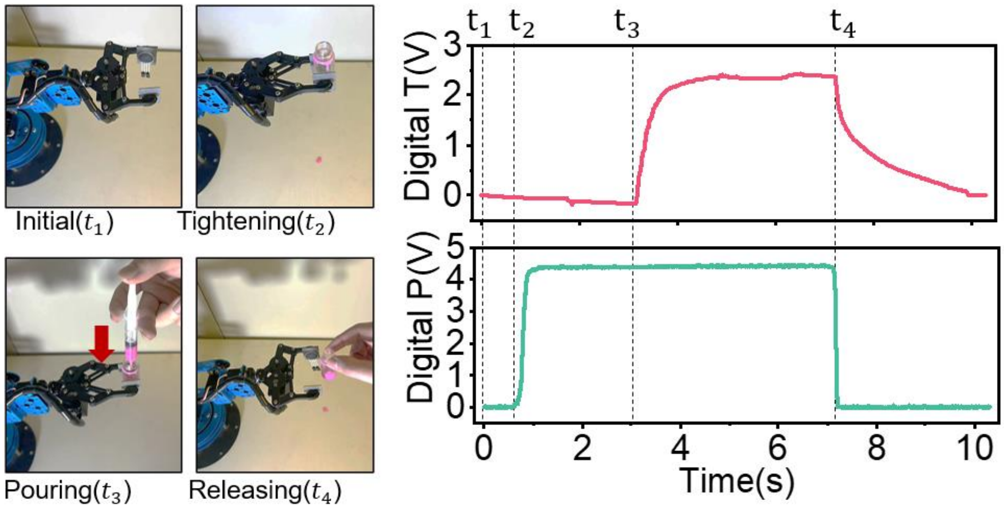

Figure S19. Photographs showing that the TPD e-skin was fixed on a robotic hand. The dual electrical responses of the TPD e-skin for the complex tactile motion.
